# APAview: A web-based platform for alternative polyadenylation analyses in hematological cancers

**DOI:** 10.3389/fgene.2022.928862

**Published:** 2022-08-12

**Authors:** Xi Hu, Jialin Song, Jacqueline Chyr, Jinping Wan, Xiaoyan Wang, Jianqiang Du, Junbo Duan, Huqin Zhang, Xiaobo Zhou, Xiaoming Wu

**Affiliations:** ^1^ The Key Laboratory of Biomedical Information Engineering of Ministry of Education, School of Life Science and Technology, Xi’an Jiaotong University, Xi’an, China; ^2^ Center for Computational Systems Medicine, School of Biomedical Informatics, University of Texas Health Science Center, Houston, TX, United States

**Keywords:** alternative polyadenylation, post-transcriptional regulation, hematological cancer, data exploration, data visualization, Flask framework, web interface

## Abstract

**Background:** Hematologic malignancies, such as acute promyelocytic leukemia (APL) and acute myeloid leukemia (AML), are cancers that start in blood-forming tissues and can affect the blood, bone marrow, and lymph nodes. They are often caused by genetic and molecular alterations such as mutations and gene expression changes. Alternative polyadenylation (APA) is a post-transcriptional process that regulates gene expression, and dysregulation of APA contributes to hematological malignancies. RNA-sequencing-based bioinformatic methods can identify APA sites and quantify APA usages as molecular indexes to study APA roles in disease development, diagnosis, and treatment. Unfortunately, APA data pre-processing, analysis, and visualization are time-consuming, inconsistent, and laborious. A comprehensive, user-friendly tool will greatly simplify processes for APA feature screening and mining.

**Results:** Here, we present APAview, a web-based platform to explore APA features in hematological cancers and perform APA statistical analysis. APAview server runs on Python3 with a Flask framework and a Jinja2 templating engine. For visualization, APAview client is built on Bootstrap and Plotly. Multimodal data, such as APA quantified by QAPA/DaPars, gene expression data, and clinical information, can be uploaded to APAview and analyzed interactively. Correlation, survival, and differential analyses among user-defined groups can be performed via the web interface. Using APAview, we explored APA features in two hematological cancers, APL and AML. APAview can also be applied to other diseases by uploading different experimental data.

## 1 Introduction

In the diagnosis and treatment of hematological cancers, genetic abnormalities at different levels have been used as biomarkers for subtyping, prognosis, and targeted therapy. 1) At a genomic level, typical abnormalities in the chromosomal karyotype and structure, such as gene fusions, structural variants, and copy number variants, have been shown to be correlated with the occurrence and development of hematological malignancies (Kerbs et al., 2021). Somatic mutations of genes such as CHEK2 and DDX41 have also been found to be pathogenic in hematological cancers (Yang et al., 2021). 2) At a transcriptional level, RNA sequencing technology has revealed that gene expression changes in hematological malignancies are related to drug resistance. For example, the high expression of ITGB2 is accompanied by high drug resistance and poor prognosis in acute myeloid leukemia (AML) (Wei et al., 2021). Using machine learning methods, it is possible to build assistant systems for the accurate diagnosis and treatment of hematological malignancies based on key genes (Lee et al., 2021). Recent studies on post-transcriptional mechanisms have shown that alterations in splicing, RNA editing, and m6A modification were found in hematological cancers (Alessandro et al., 2000; Li et al., 2017).

Alternative polyadenylation (APA) is an important post-transcriptional regulation process that often occurs in introns or 3′UTR to form the 3′ end of mature mRNA. APA in the intronic region leads to truncation of mature mRNA. In chronic lymphocytic leukemia, loss of DICER and FOXN3 functions was due to intronic APAs which led to truncated protein products (Lee et al., 2018). APA occurring in 3′UTR changes the length of 3′UTR, which affects mRNA stability, subcellular localization, and translation efficiency (Zhang et al., 2021). The advancements in transcriptomic sequencing (e.g., 3′RACE, PolyA-Seq, and TRENDseq (Ogorodnikov and Danckwardt, 2021)) have allowed for the establishment of APA and polyadenylation site signal (PAS) databases such as polyA DB3 (Wang et al., 2018), APAatlas (Hong et al., 2020), and TREND-DB (Marini et al., 2021). In addition, algorithms such as QAPA (Ha et al., 2018) and DaPars (Xia et al., 2014) were developed to identify and quantify APA based on RNA sequencing data.

With the discovery of APA differences across tissues, more and more studies have focused on identifying APA roles in cancers. Using TCGA RNA sequencing data, TC3A (Feng et al., 2018) calculated the percentage of distal PolyA site usage index (PDUI) using DaPars and found a widespread shortening of 3′UTR across cancers. SNP2APA used PDUIs as quantitative traits to analyze effects of SNPs on APA in different TCGA tumors (Yang et al., 2020). In hematological malignancies, the APA regulator FIP1L1 was found to block cell differentiation (Davis et al., 2021). Through an analysis of AML single-cell RNA sequencing data, researchers have found APA dynamics in different cell types, suggesting a potential relationship between APA and hematological cell abnormalities (Ye et al., 2019). These studies have provided new avenues for hematological cancer intervention from the level of post-transcriptional modification.

The different output formats of current RNA-sequencing-based APA identification tools have made it difficult to obtain reliable APA loci from multiple methods for subsequent analysis. Additional data preprocessing is needed to unify the results, which could be complicated, time-consuming, and laborious. In order to facilitate the study of APA effects on hematological malignancies, we developed a web-based platform called APAview to analyze APA data from QAPA and DaPars. The platform can: 1) provide correlation analysis between the APA usage index and gene/transcript expression; 2) compare the APA usage index and gene/transcript expression differences between groups; 3) identify genes with shortened/lengthened 3′UTR between groups based on the APA usage index; 4) carry out APA-based survival analysis; 5) annotate genes and APA sites using databases such as UCSC and polyA DB3; and 6) visualize gene structures, APA sites, and related motifs. All results can be downloaded from the APAview web pages.

## 2 Methods

**FIGURE 1:**
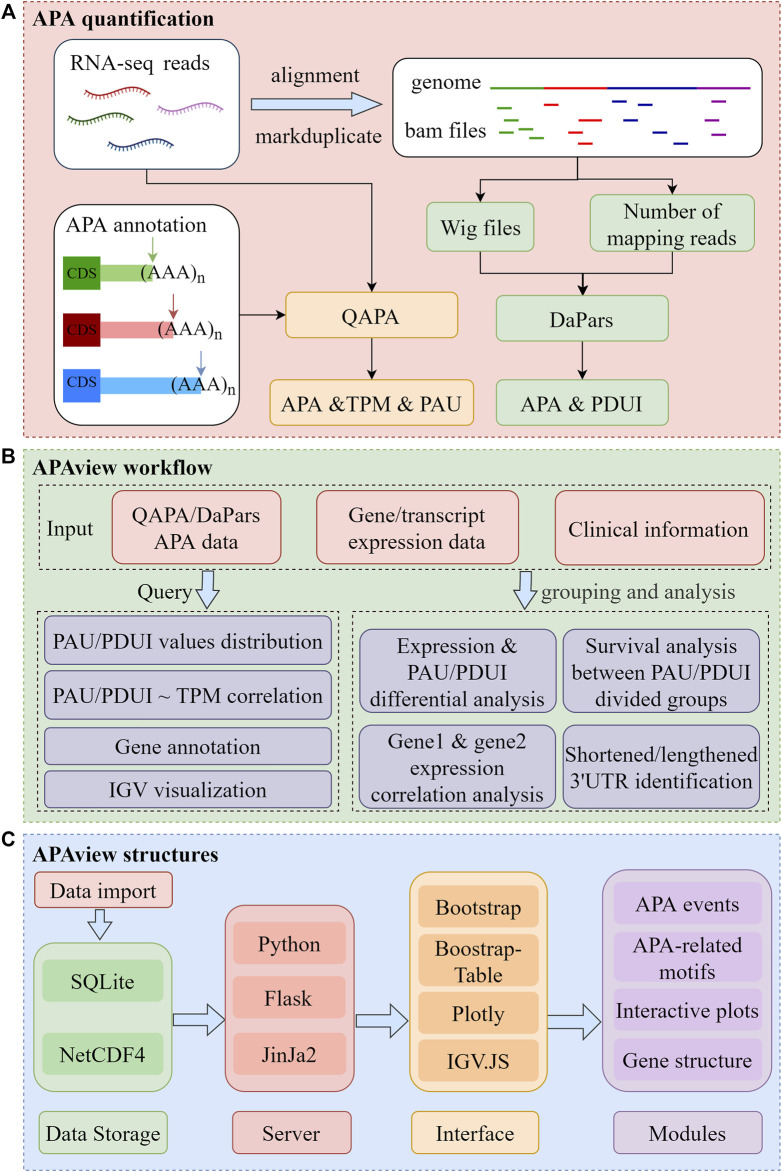
Flow chart of APAview. **(A)** Data preprocessing for APA quantification. Taking QAPA and DaPars as examples, APA quantification processes taking RNA sequencing raw data as input are shown. QAPA uses APA site annotation in PolyA site and GENCODE to construct transcripts 3′UTR and calculate TPM and PAU. DaPars takes wig files produced after alignment as input, normalizes library sizes using the number of mapping reads, and calculates PDUI for each gene. **(B)** Query and functional analysis of APA. Taking APA quantification, gene/transcript expression, and clinical data as input, APAview provides APA query and visualization in the Query module. In the Analysis module, APAview achieves correlation analysis, survival analysis, differential analysis, and lengthening/shortening gene identification. Features between user-defined groups can be compared. **(C)** Storage and display structures of APAview. APAview stores data in SQLite and NetCDF4 files. Python, Flask, and Jinja2 are used to build the interactive web platform. APA data are shown as tables using Bootstrap-Table; analysis results are shown using Plotly; gene structure and APA-related motifs are shown using IGV. JS. PAU, PolyA site usage; PDUI, percentage of distal PolyA site usage index; TPM, transcripts per kilobase million.

APAview consists of three major sections, as depicted in Figure 1. First, APAs are quantified using QAPA and DaPars. Second, APAs can be queried and analyzed in APAview. Third, data and analysis results are visualized. The workflow is described in detail in Sections 2.1–2.4.

### 2.1 Data preprocessing

APAview first extracts 3′UTR APA position and usage information from QAPA and DaPars. The basic process for producing appropriate input to APAview in the human genome using RNA sequencing raw data (Fastq files) is shown in Figure 1A, which runs on Linux.

For RNA sequencing data, alignment tools such as HISAT2 (Kim et al., 2015) and STAR2 (Dobin et al., 2013) map reads to human hg19/hg38 reference genome. PCR duplicates generated during sequencing can be removed from Bam files using GATK MarkDuplicate (McKenna et al., 2010) or sambamba markdup (Tarasov et al., 2015) commands.

In the quantification of APA usage, QAPA uses Salmon (Patro et al., 2017) to quantify the expression of 34,978 transcripts with PolyASite database (Herrmann et al., 2020) and GENCODE APA sites annotation (Harrow et al., 2006). Then, PolyA site usage (PAU) is calculated as the proportion of the transcript expression among the gene, which ranges from 0 to 1. A PAU closer to 1 represents more transcripts using the APA site, and a PAU closer to 0 represents less APA site usage. In addition to Bam files, QAPA also accepts Fastq files as input for APA quantification. The output contains information on gene IDs, gene symbols, transcript IDs, chromosomes, APA IDs, start and end position of 3′UTR and last exons, length of 3′UTR, number of APA sites, transcripts per kilobase million (TPM) expression, and PAU, among which the expression column is identified by “TPM” suffix and the PAU column by “PAU” suffix.

Compared with QAPA, DaPars does not depend on APA site annotation. It uses a regression model to identify and quantify dynamic APA events in a set of samples and only keeps one major proximal APA site for one transcript. DaPars measures the usage proportion of distal APA sites (or PDUI), which also ranges from 0 to 1. A value of 1 indicates that all transcripts of the gene use the distal APA site instead of the proximal site. DaPars uses Wig files as input and corrects APA quantitative results based on the alignment depth, which can be obtained from Bam files using the BEDTools genomeCoverageBed (Quinlan and Hall, 2010) command and SAMtools flagstat (Li et al., 2009) command, respectively. The output information of DaPars includes columns of chromosomes, gene ID, APA sites, 3′UTR, and PDUI. Since the results of DaPars do not contain transcript expression, they can be inferred by using featureCounts (Liao et al., 2014) or Salmon.

In order to extend the usage of APAview, we also allow users to upload APA data identified by other methods. The acceptable APA data format is described in detail on the “Help” page of APAview.

### 2.2 Analysis of alternative polyadenylation data

APAview integrates APA, gene expression, and clinical data to provide data query and analysis functions for convenient APA feature mining in hematological cancers (Figure 1B). Specifically, APAview provides correlation analysis, survival analysis, differential analysis, and lengthening/shortening gene identification modules to study APA differences between user-defined groups and their effects on gene expression.

Differential APA usage can indicate differential 3′UTR lengths. Therefore, APAview provides a differential module to compare PAU/PDUI among groups through the Mann–Whitney test or Kruskal–Wallis test. PAU/PDUI distribution is shown in a density plot. The trend of 3′UTR lengthening and shortening caused by APA is represented by ΔPAU/ΔPDUI , which is calculated as the difference between the mean value of PAU/PDUI in two groups. Also, the differential module could compare gene expression between groups. Differential analysis results are displayed in box plots.

Correlation analysis in APAview is used to study the linear relationship between APA indexes and gene expression. It can be performed for all samples, or within user-defined groups. In addition, we provide correlation analysis between expressions of the two specified genes to evaluate regulation between genes. The analysis results are interactively visualized using dot plots and regression analysis curves with a 95% confidence interval.

Survival analysis is commonly used to assess relationships between molecular features and prognosis. APAview provides survival analysis based on APA indexes. For a specific gene, the median of the APA index is used as a threshold to divide samples into two groups. Survival comparison between groups is implemented using the log-rank method and visualized using KM curves.

### 2.3 Web server design

APAview is a web-based system developed using open-source technologies (Figure 1C). The server runs on Python3 (https://www.python.org/), Jinja2 (https://jinja.palletsprojects.com/), and Flask (https://flask.palletsprojects.com/) for the query and analysis of APA data. The browser aspect is built upon Bootstrap (https://getbootstrap.com/), Bootstrap-Table (https://bootstrap-table.com/), and Plotly (https://github.com/plotly/plotly.py). It provides the user interface for visualizing and interacting with APA information and analysis results. Statistical methods such as linear regression, Mann–Whitney test, Kruskal–Wallis test, and survival analysis are implemented in Python using the libraries NumPy (Harris et al., 2020), SciPy (Virtanen et al., 2020), and Statsmodels (Seabold and Perktold, 2010). An SQLite database (https://www.sqlite.org) is used to store APA site information. The NetCDF4 format file is used to store expression data and read to the APAview server by the library xarray (Hoyer and Hamman, 2017). A genome browser based on IGV. JS (Robinson et al., 2011) is integrated into APAview to display gene structure, APA sites, and molecular features.

### 2.4 Workflow

**FIGURE 2:**
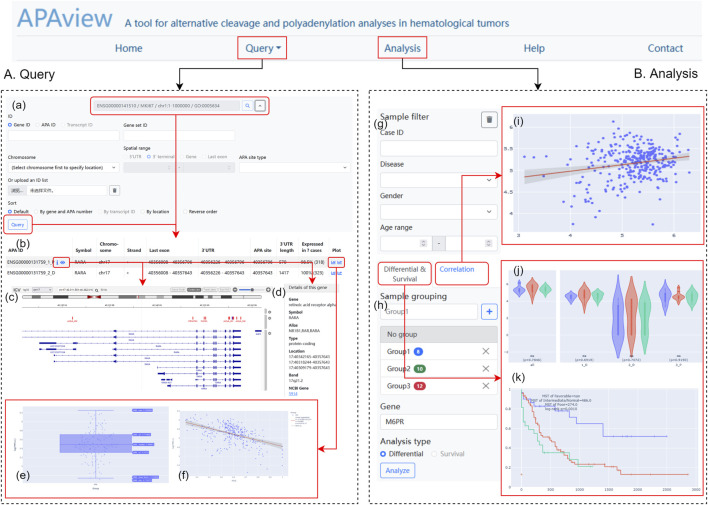
Web interface of APAview. **(A)** On the “Query” page, gene/transcript symbols can be inputted **(a)**, and the retrieved APA information is shown in table **(b)**. APA sites, 3′UTR, PASs, RBP-, miRNA-binding, and gene structures are shown by IGV **(c)**. Gene annotation from NCBI is provided (d). PAU/PDUI distribution **(e)** and the correlation between PAU/PDUI and gene expression **(f)** are shown in interactive plots. **(B)** On the “Analysis” page, samples could be filtered **(g)** and grouped **(h)** based on clinical information. Results of correlation **(i)**, differential **(j)**, and survival analyses **(k)** are shown in interactive plots. PAS, PolyA signal; RBP, RNA-binding protein; PAU, PolyA site usage; PDUI, percentage of distal PolyA site usage index.

APAview provides a user-friendly web interface for APA query, analysis, and display. By compressing APA, expression, and clinical data into a file, APAview produces an SQLite database via script and uploads it to the server. APAview provides a “Query” page for APA details and visualization, and an “Analysis” page for APA-related analysis (Figure 2).

On the “Query” page (Figure 2A), users can retrieve APA information using fields such as gene ID, gene name, and transcript ID. Since APAview provides built-in ID mappings, ID types that are not contained in raw data can also be used as search keys. In advanced search, APAview also accepts gene lists and specific ranges in chromosomes. Annotated information on genes from NCBI (https://www.ncbi.nlm.nih.gov/) is provided along with gene IDs. The scatter diagram and regression curve in the “Plot” column show the correlations between PAU/PDUI and gene expression in all samples. IGV plots contain six tracks that display gene structures and molecular features, including APA sites and 3′UTR extracted from the input data, PASs from polyA DB3 database, RBP from ENCODE (Luo et al., 2020), miRNA from TargetScanHuman 7.2 (Agarwal et al., 2015), and human hg19/hg38 genome.

On the “Analysis” page (Figure 2B), users can group samples based on clinical information and apply differential, correlation, and survival analyses. Clinical data is displayed in a “Case table” and users can show and hide columns of interest. Box plots, dot plots, and KM curves are displayed on the site and are downloadable. All plots have legends to label groups and show statistical results. Users can interactively examine data summary information, check samples’ information, and choose visible elements on plots.

In addition, the “Help” page provides instructions for using the APAview platform. Any questions and suggestions can be reported through the email address on the “Contact” page.

## 3 Results

Two hematological cancer datasets were analyzed to illustrate the usage of APAview. The first dataset was downloaded from GSE172057 (Lin et al., 2021). This dataset contained RNA sequencing data for 323 acute promyelocytic leukemia (APL) patients. QAPA was used to quantify APA usage, and the PAU was calculated as an APA index. Clinical information such as age, gender, and prognostic risk was collected from the original research. The second dataset is from the TC3A database (http://tc3a.org). APA data of 144 AML samples were quantified by DaPars. Gene expression and clinical information were downloaded from TCGA and were analyzed in conjunction with APA data in APAview.

### 3.1 Acute promyelocytic leukemia case study

The stratification of APL is mainly based on white blood cell count and platelet count (Lin et al., 2021). Precise molecular markers may help reduce early death and recurrence of patients. The original study of the APL dataset constructed an APL stratification index and reported 155 mutated genes associated with patient prognosis. Using APAview, these APL genes were assessed at a post-transcriptional level.

**FIGURE 3:**
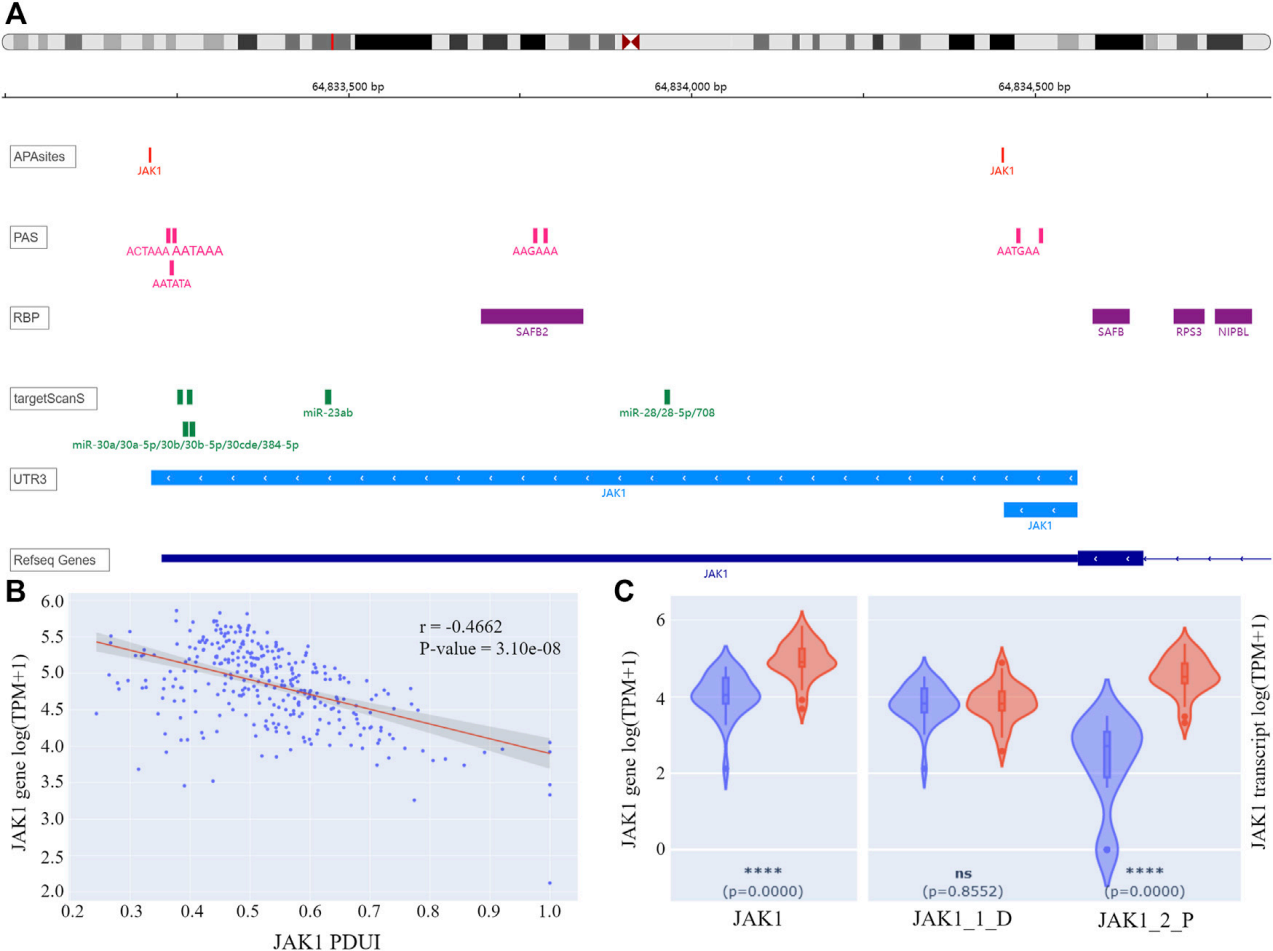
**(A)** IGV plot of JAK1. The tracks from top to bottom are APA sites extracted from input data, PASs annotated by polyA DB3, RBP-binding regions annotated by ENCODE, miRNA-binding regions annotated by TargetScanHuman 7.2, 3′ UTR constructed by APA sites, and human genome from UCSC. **(B)** Correlation analysis result of JAK1 PDUI and gene expression. The X-axis represents PDUI of the distal JAK1 APA site; the Y-axis represents the expression of JAK1. Each dot in the scatter plot represents an APL sample. The red line is the regression curve, and the gray region shows its 95% confidence interval. **(C)** Box plots of gene and transcript expression of JAK1 in the high-PDUI group (blue) and low-PDUI group (red). “JAK1_1_D” represents the transcript with the distal APA, and “JAK1_2_P” represents the transcript with the proximal APA. **** *p*-value < 0.0001. ns *p*-value > 0.05. PDUI, percentage of distal PolyA site usage index; PAS, PolyA site signal; RBP, RNA-binding protein; TPM, transcripts per kilobase million.

For the 155 APL-related genes, APA information was queried by gene symbols using APAview. Eighty-two genes had APA sites constituting transcripts with 3′UTR of different lengths. For example, Janus Kinase I (JAK1) had two QAPA-annotated APA sites with coordinates chr1: 64833213 and chr1:64834454, constituting 3′UTRs with lengths of 1348bp and 107bp, respectively (Figure 3A). We defined the former as the distal APA and the latter as the proximal APA.

APAview analysis showed a negative correlation between PDUI and JAK1 expression, i.e., there was a decrease in gene expression with an increase in distal APA usage (Figure 3B). Comparing JAK1 expression between the top 30 samples and the bottom 30 samples of PDUI values, expression of the JAK1 gene was significantly less in the high-PDUI group (Figure 3C). According to polyA DB3 database information, there was a canonical PAS AATAAA located 40 nt upstream of the distal site, but there was no PAS for the proximal site, leading to a higher distal APA site usage (Figure 3A). APA cleavage at the distal site leads to the downregulation of the shorter transcript in the high-PDUI group. Biological experiments can be designed for further validation of the hypothesis.

Genes that may interact with JAK1 were extracted from the STRING database (Szklarczyk et al., 2019), and their correlation in APL was analyzed with APAview. The results showed that the expressions of genes such as STAT1, STAT3, GRB2, SOCS5, PTPN11, and MDM2 were positively correlated with JAK1 (*p*-value < 0.05) (Table1, Supplementary Figures S1–S14). These interacted genes were enriched in the JAK-STAT signaling pathway through KEGG pathway enrichment analysis. Previous studies have shown that the activation of the JAK-STAT pathway is required to induce differentiation of the APL cell line HT93A (Uchino et al., 2015). Therefore, the decreased expression of JAK1 through decreasing proximal APA usage might lead to cell differentiation block and affect the occurrence and development of APL.

**TABLE 1:** Gene expression correlation between JAK1 and STRING-reported genes in APL.

Gene symbol	Description	r	*p*-value
STAT3	Signal transducer and activator of transcription 3	0.6853	3.95e-46
IL6ST	Interleukin 6 cytokine family signal transducer	0.3677	8.87e-12
MDM2	MDM2 proto-oncogene	0.5611	3.48e-28
INSR	Insulin receptor	0.4997	8.29e-22
STAM2	Signal-transducing aaptor molecule 2	0.5357	2.21e-25
GRB2	Growth factor receptor-bound protein 2	0.6533	1.09e-40
PTPN11	Protein tyrosine phosphatase non-receptor type 11	0.5658	9.96e-29
STAT5B	Signal transducer and activator of transcription 5B	0.4360	2.04e-16
SOCS5	Suppressor of cytokine signaling 5	0.7399	3.23e-57
IL4R	Interleukin 4 receptor	0.3250	2.22e-09
STAT1	Signal transducer and activator of transcription 1	0.6954	5.54e-48
STAT5A	Signal transducer and activator of transcription 5A	0.3845	8.02e-13
CSF2RB	Colony-stimulating factor 2 receptor subunit beta	0.4402	9.74e-17
IFNGR1	Interferon gamma receptor 1	0.3459	1.65e-10

Unfortunately, the survival information for these APL samples is yet to be released, so the role of JAK1 in patients’ survival will be completed in later studies.

### 3.2 Acute myeloid leukemia case study

A previous study on APA data from TCGA samples demonstrated that CFIm25-mediated 3′UTR shortening promotes glioma growth (Masamha et al., 2014), suggesting that APA might serve as a novel prognostic biomarker. Here, we used APAview to identify potential AML-related APA features.

**FIGURE 4:**
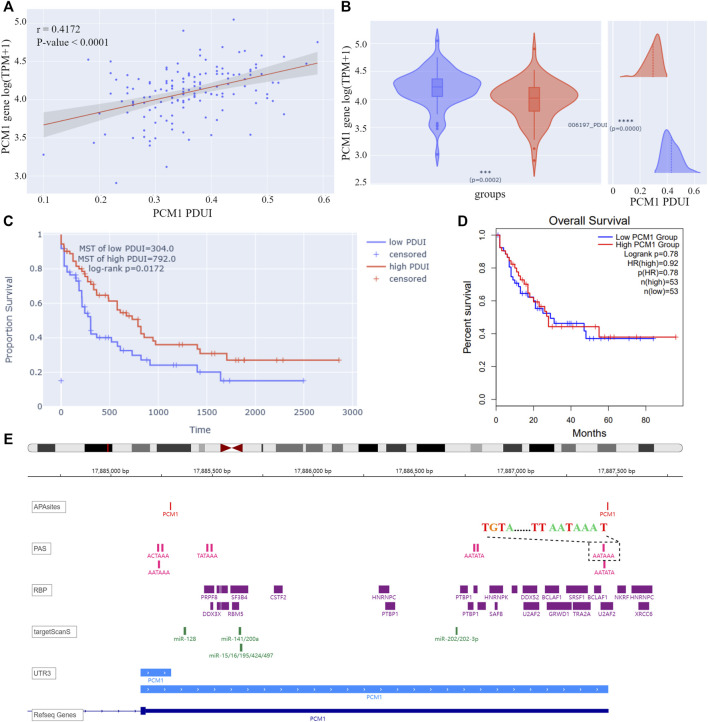
**(A)** Correlation analysis result of PCM1 PDUI and gene expression. The X-axis represents PDUI of PCM1; the Y-axis represents expression of PCM1. Each dot in the scatter plot represents an AML sample. The red line is the regression curve, and the gray region shows its 95% confidence interval. **(B)** Box plot of PCM1 expression and density plot of PCM1 PDUI in the high-PDUI group (blue) and low-PDUI group (red). Dashed lines represent mean values of PDUI in groups. *** *p*-value < 0.001, **** *p*-value < 0.0001. **(C)** KM curve of PCM1 low-PDUI and high-PDUI groups in AML. Groups are divided by the median of PCM1 PDUI. Survival time is shown by days. **(D)** KM curve of PCM1 low-expression and high-expression groups in AML. Groups are divided by the median of PCM1 expression in AML. The plot is downloaded from GEPIA2. Survival time is shown by months. **(E)** IGV plot of PCM1. The tracks from top to bottom are APA sites extracted from input data, PAS annotated by polyA DB3, RBP-binding regions annotated by ENCODE, miRNA-binding regions annotated by TargetScanHuman 7.2, 3′ UTR constructed by APA sites, and human genome from UCSC. The dashed box showed the PAS sequence upstream of the distal APA site of PCM1. PDUI, percentage of distal PolyA site usage index; MST, median survival time; PAS, PolyA site signal; RBP, RNA-binding protein; TPM, transcripts per kilobase million; HR, hazard ratio.

On the “query” page, APA information for 209 AML-related genes was queried. Twelve genes were found to have significant correlations between PDUIs and gene expression (|r| > 0.3 & *p*-value < 0.05), among which the expression of pericentriolar material 1 (PCM1) increased with the lengthening of 3′UTR of gene transcript NM_006197 (Figure 4A). AML samples were divided into two groups based on their PDUI of PCM1. Using the differential analysis modules on the “Analysis” page, PCM1 was found to be overexpressed in the high-PDUI group (Figure 4B). Patients in the high-PDUI group had better survival rates (Figure 4C). In contrast, TCGA AML patients did not show a survival difference between groups divided using the median of PCM1 expression in GEPIA2, a web server for expression analysis of TCGA data (Figure 4D) (Tang et al., 2019). This indicates a potential prognostic role of PCM1 APA site preference in AML.

According to cleavage motifs annotated in polyA DB3, there is an AATAAA motif 40 nt upstream of the PCM1 proximal APA site, and AATAAA and TGTA elements 100 nt upstream of the distal APA site (Figure 4E). APAview showed interesting correlations between PCM1 and 11 cleavage and polyadenylation factors (Table 2, Supplementary Figures S15–S25). Among which, CPSF2, CPSF3, FIP1L1, and WDR33 are subunits of cleavage and polyadenylation specificity factor (CPSF); CSTF1, CSTF2, CSTF3, and CSTF2T are part of the cleavage stimulation factor (CSTF); CPSF6 and NUDT21/CPSF5 constitutes the cleavage factor I (CFI); and PCF11 is a subunit of cleavage factor II (CFII). CPSF interacts with the AATAAA motif through FIP1L1 and WDR33. CFI, in conjunction with NUDT21, binds to the TGTA element (Tian and Manley, 2017). TGTA elements were found around the APA sites of PCM1. A previous study has reported that FIP1L1 might be an important mediator of APA alterations in AML (Davis et al., 2018). The analysis results from APAview suggest a relationship between APA factors and PCM1 APA site preference. Additional studies are needed to validate this relationship.

**TABLE 2:** Gene expression correlation between PCM1 and APA regulatory factors in AML.

Gene symbol	Description	r	*p*-value
CPSF2	Cleavage and polyadenylation specific factor 2	0.5432	2.00e-12
CPSF3	Cleavage and polyadenylation specific factor 3	0.6081	6.28e-16
CPSF6	Cleavage and polyadenylation specific factor 6	0.6356	1.15e-17
CSTF1	Cleavage stimulation factor subunit 1	0.5035	1.26e-10
CSTF2	Cleavage stimulation factor subunit 2	0.4697	2.86e-09
CSTF3	Cleavage stimulation factor subunit 3	0.6124	3.47e-16
PCF11	PCF11 cleavage and polyadenylation factor subunit	0.5538	6.03e-13
CSTF2T	Cleavage stimulation factor subunit 2 Tau variant	0.6041	1.10e-15
WDR33	WD repeat domain 33	0.6508	1.05e-18
FIP1L1	Factor interacting with PAPOLA and CPSF1	0.6318	2.05e-17
NUDT21	Nudix hydrolase 21	0.6919	7.90e-22

## 4 Discussion

The increase of data availability for hematological cancers provides a large repertoire of RNA sequencing data that can be used to find prognostic markers, therapy targets, and classification indicators. Although allogeneic stem cell transplant, chemotherapy, immunotherapy, hypomethylating agents, and targeted small molecules have been widely used in the treatment and subsequent post-remission therapy of hematological cancers, high relapse rates and drug resistance are still tricky challenges to prolonging survival time, especially for patients at high risk. Efficient biomarkers identified from different genetic levels are urgently needed. In this study, we introduce APAview, a web-based platform that allows the analysis of post-transcriptional APA features in hematological malignancies. Users can upload data to APAview and perform APA-related information retrieval, analysis, and visualization. The ability to define user-based analyses increases the flexibility and freedom of the platform. To our knowledge, APAview is the first interactive tool focused on APA features mining.

As shown by two case studies, APAview can be used to rapidly reveal relationships between APA and gene expression, patient survival, and molecular binding. The entire process can be performed interactively with a web interface without any additional scripts. At present, APAview can automatically recognize fields from outputs of QAPA and DaPars, but data from other methods are also accepted. Its seamless compatibility with other methods will be developed in future versions. In addition, modules for batch processing of multi-genes will be added for more general analyses. Since there are many algorithms for APA quantification, and most of them are time-consuming and memory-consuming, we did not integrate the preprocessing in APAview; however, we provide guidelines and resources for users to quantify APA.

In conclusion, APAview specializes in the analyses of APA features in diseases, simplifies the process of APA data mining, and provides interactive visualization results. The study of APA features will contribute to the discovery and practice of disease intervention at the RNA level and improve the understanding of pathogenic mechanisms.

## Data Availability

APAview is fully open-sourced and freely accessible from https://github.com/Wu-xjtu/APAview. The datasets presented in this study can also be found at this link.
