# Supplementary material for: APAview: A web-based platform for alternative polyadenylation analyses in hematological cancers
Source: Front Genet. 2022 Aug 12;13:928862. doi: 10.3389/fgene.2022.928862 (PMC9411867; doi:10.3389/fgene.2022.928862)
Supplement: Supplementary file 1 [file DataSheet1.docx]

Supplementary material


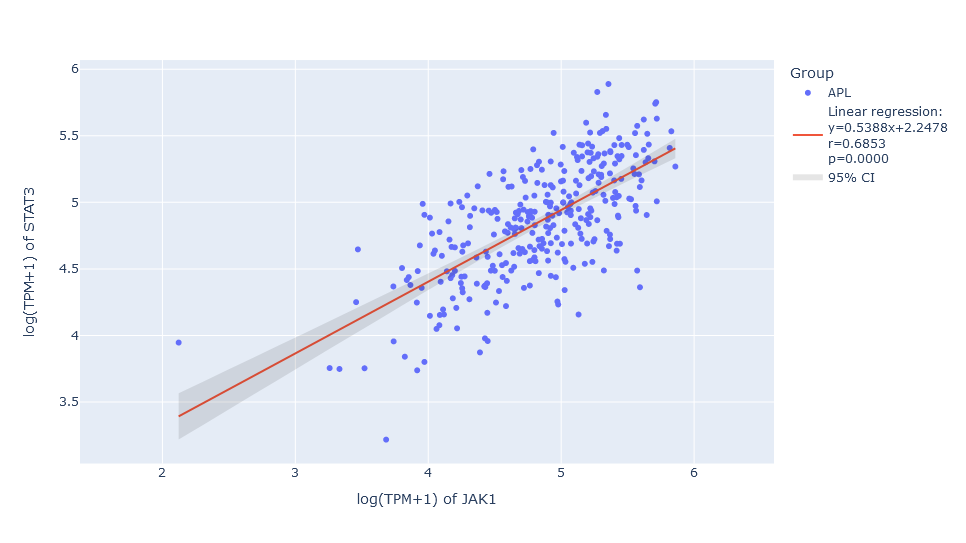


Figure S1. Correlation analysis results of expression of JAK1 and STAT3, r = 0.6853, P-value = 3.95e-46.


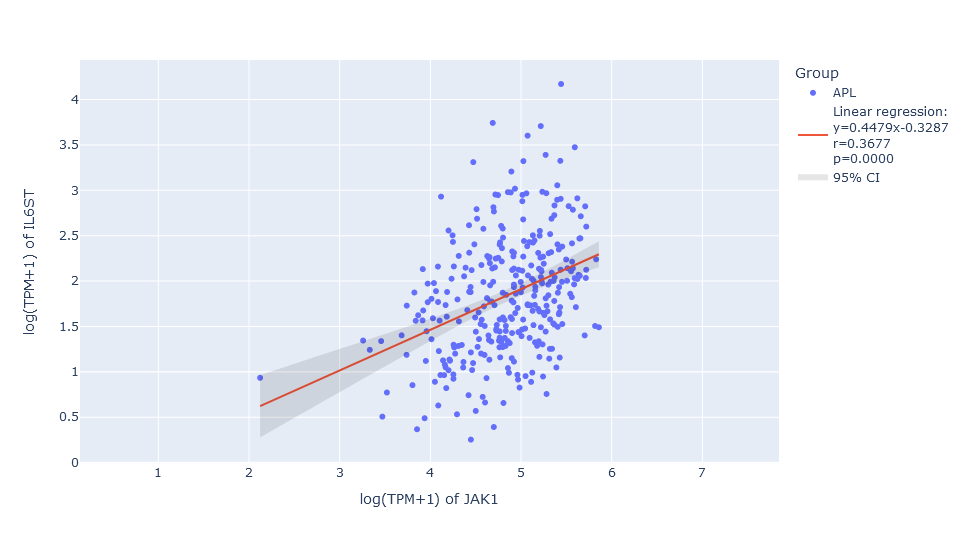


Figure S2. Correlation analysis results of expression of JAK1 and IL6ST, r = 0.3677, P-value = 8.87e-12.


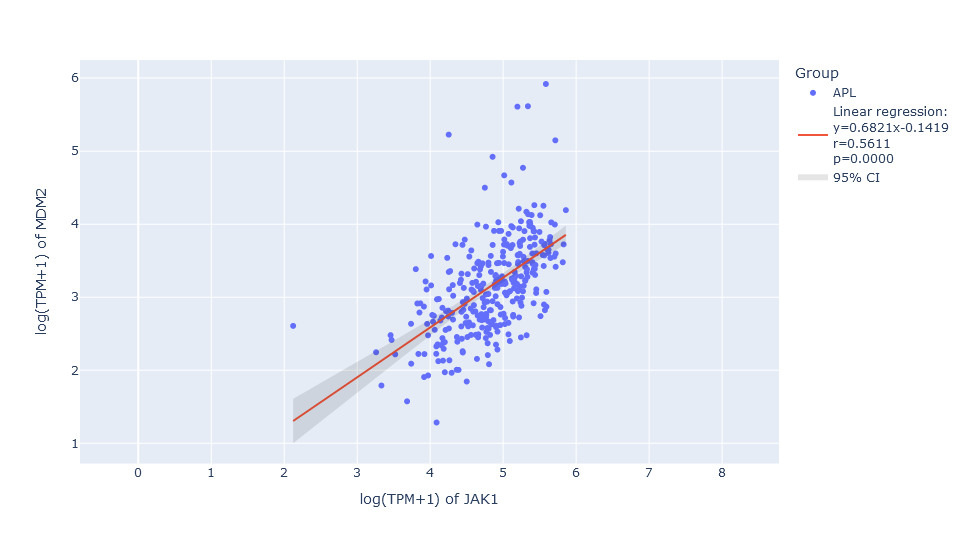


Figure S3. Correlation analysis results of expression of JAK1 and MDM2, r = 0.5611, P-value =3.48e-28.


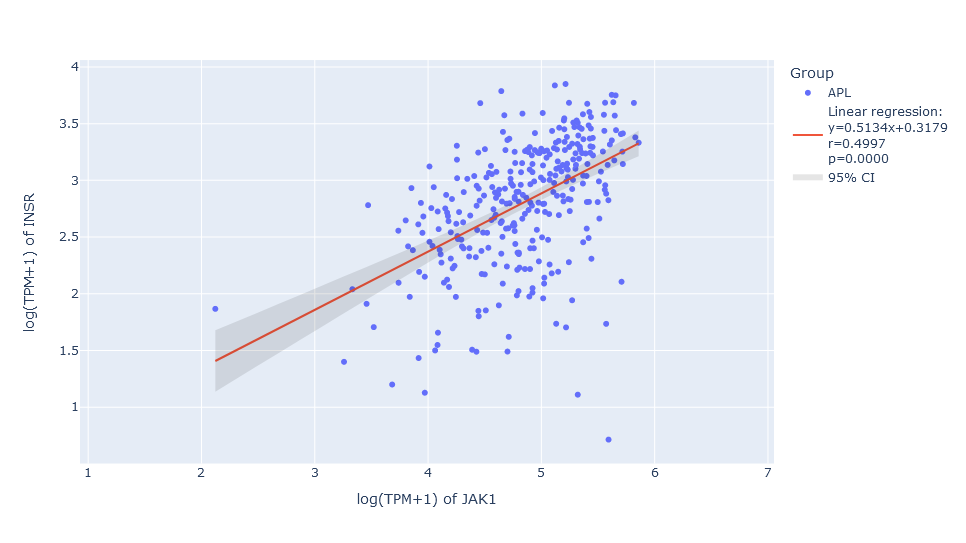


Figure S4. Correlation analysis results of expression of JAK1 and INSR, r = 0.4997, P-value = 8.29e-22.


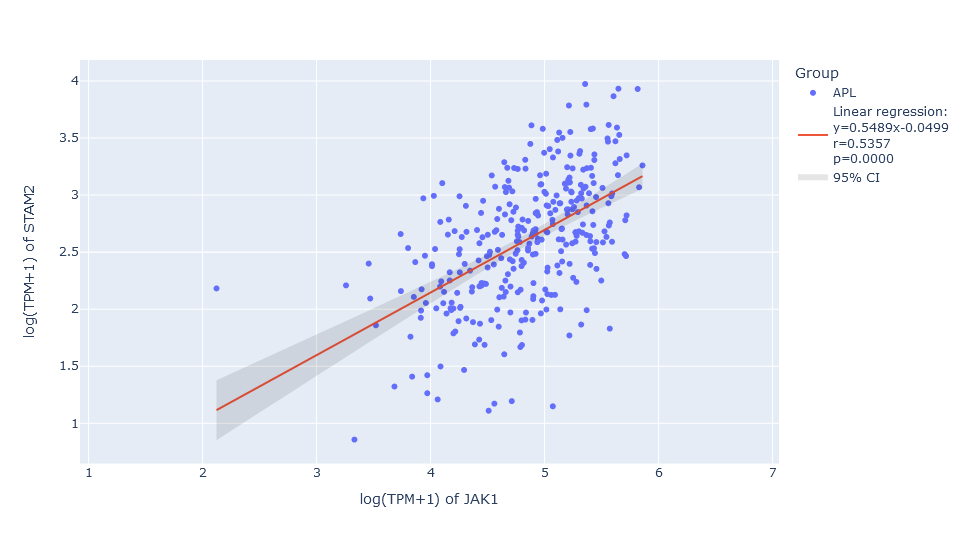


Figure S5. Correlation analysis results of expression of JAK1 and STAM2, r = 0.5357, P-value = 2.21e-25.


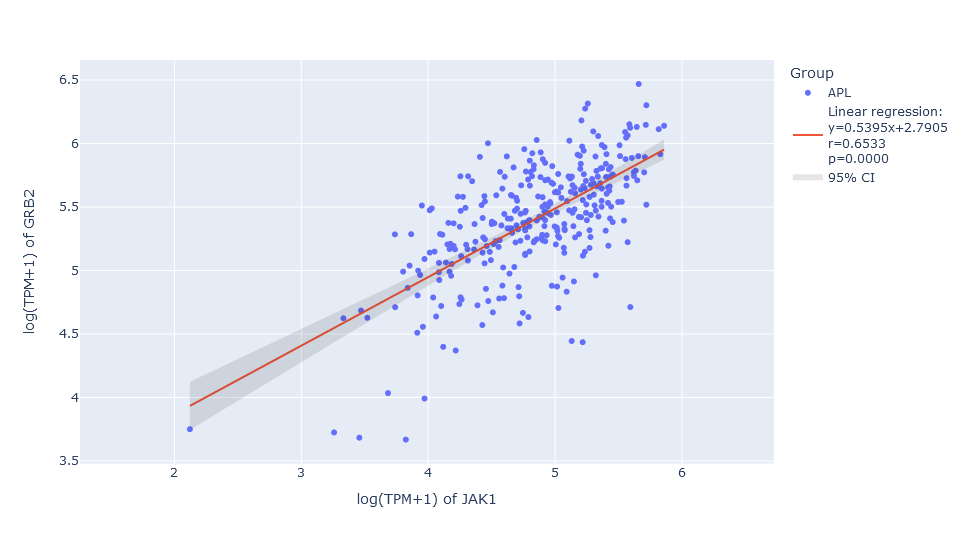


Figure S6. Correlation analysis results of expression of JAK1 and GRB2, r = 0.6533, P-value = 1.09e-40.


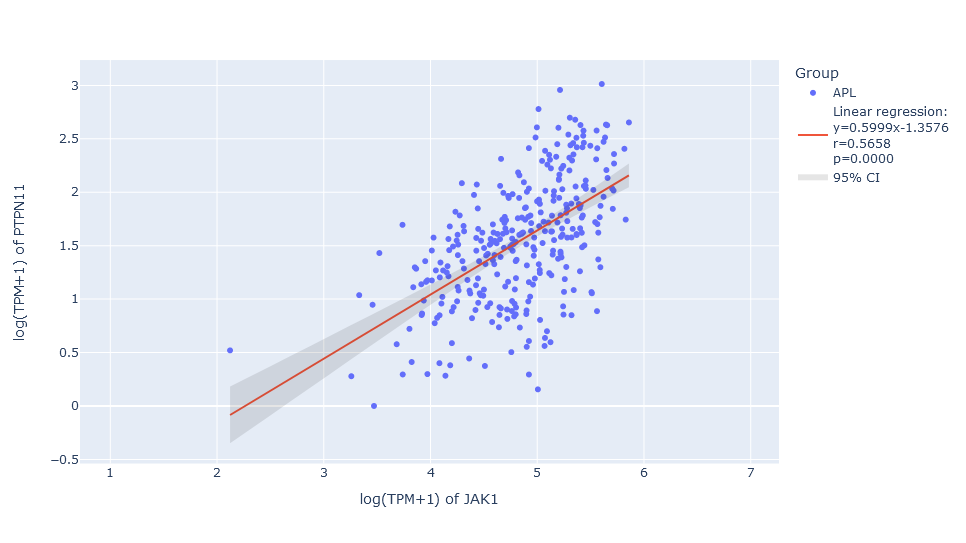


Figure S7. Correlation analysis results of expression of JAK1 and PTPN11, r = 0.5658, P-value = 9.96e-29.


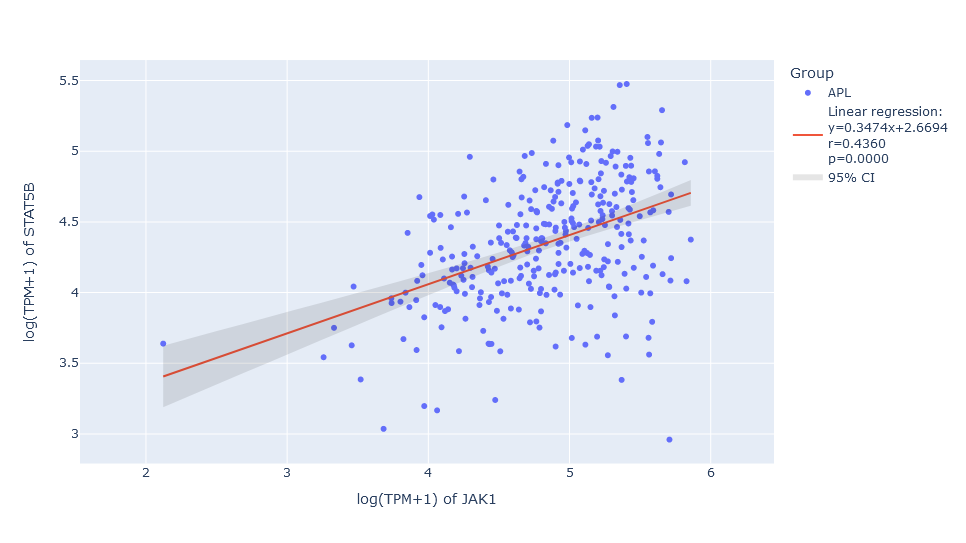


Figure S8. Correlation analysis results of expression of JAK1 and STAT5B, r = 0.4360, P-value =2.04e-16.


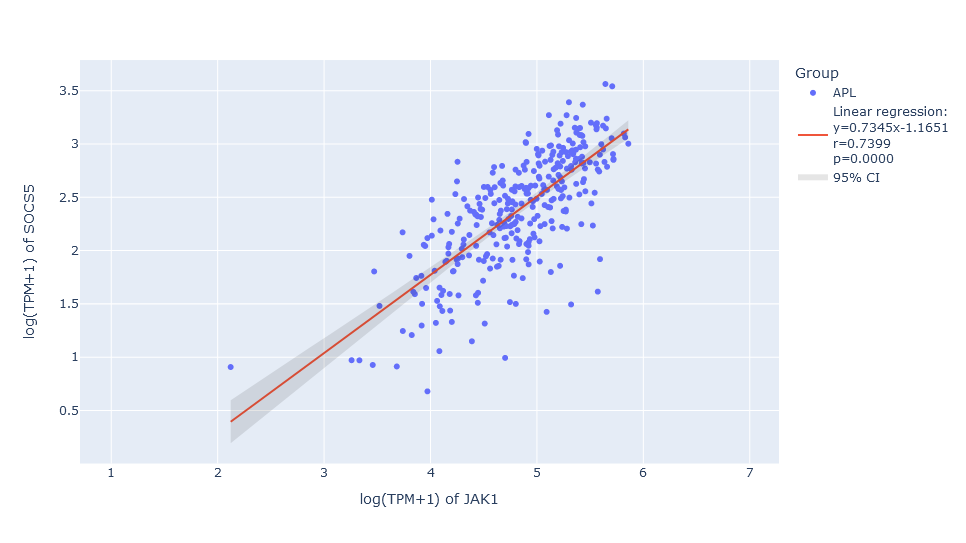


Figure S9. Correlation analysis results of expression of JAK1 and SOCS5, r = 0.7399, P-value =3.23e-57.


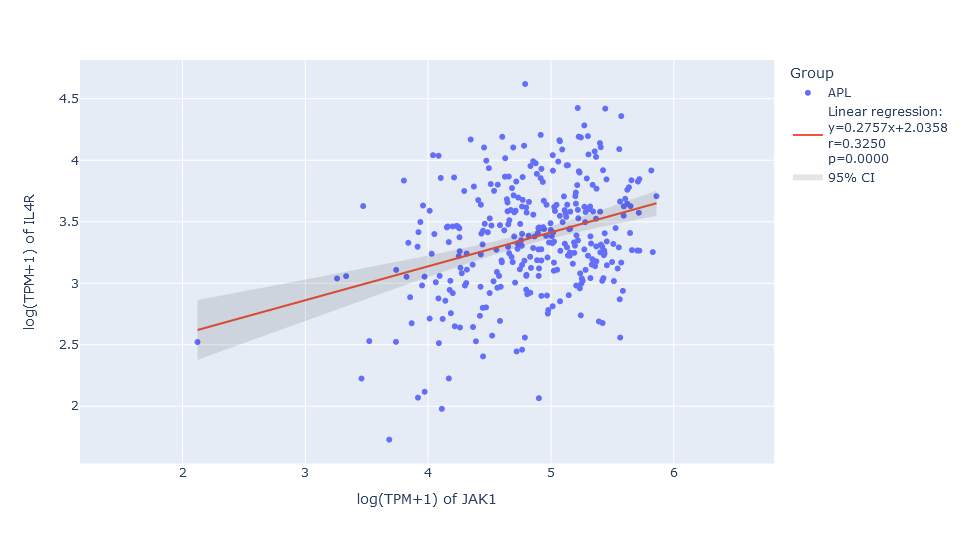


Figure S10. Correlation analysis results of expression of JAK1 and IL4R, r = 0.3250, P-value =2.22e-09.


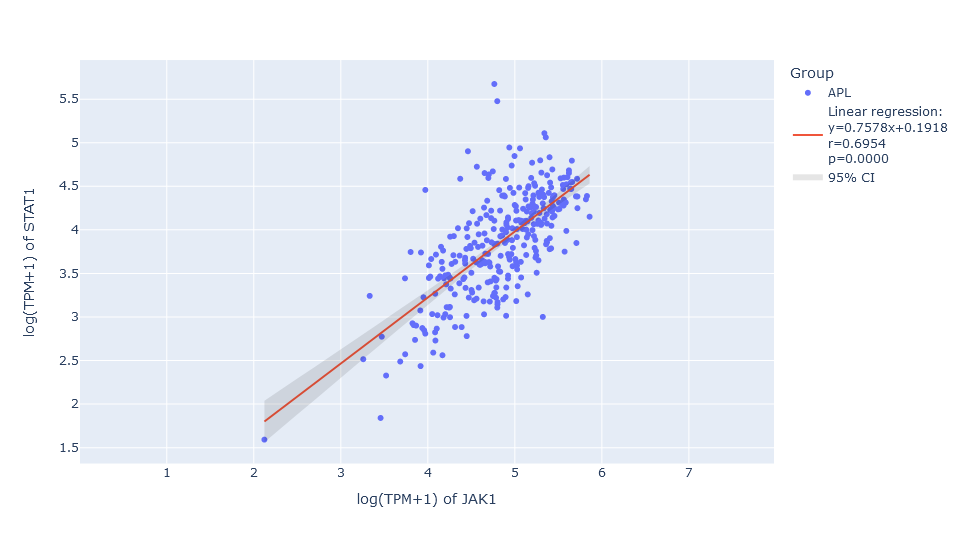


Figure S11. Correlation analysis results of expression of JAK1 and STAT1, r = 0.6954, P-value =5.54e-48.


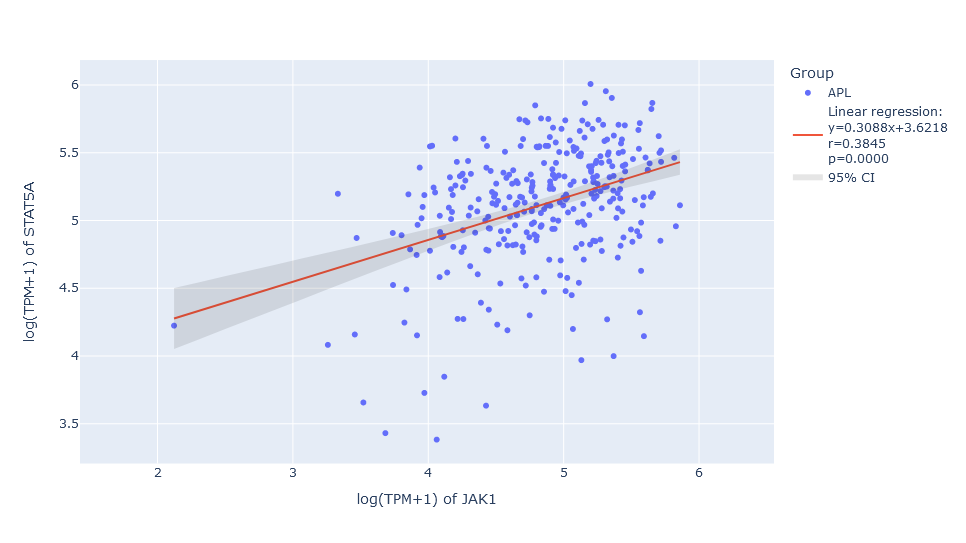


Figure S12. Correlation analysis results of expression of JAK1 and STAT5A, r = 0.3845, P-value =8.02e-13.


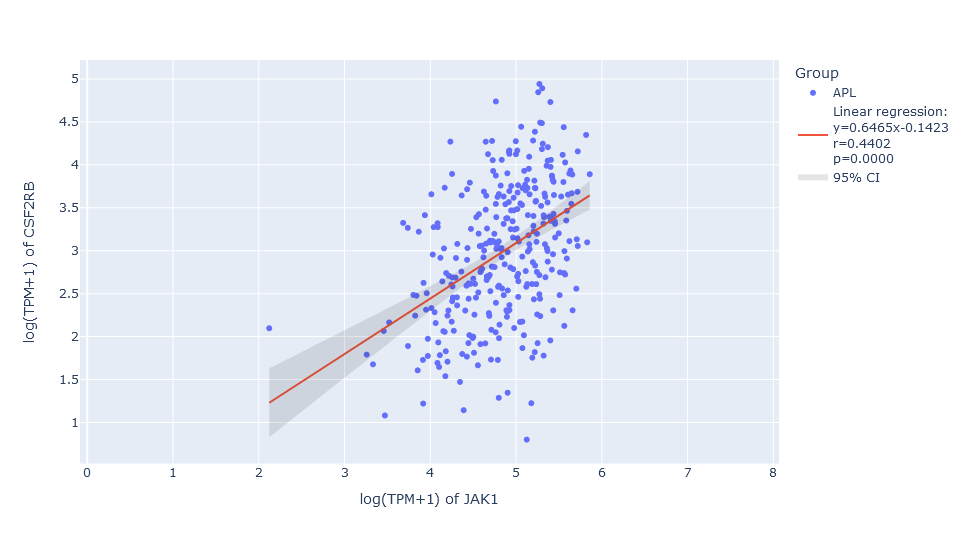


Figure S13. Correlation analysis results of expression of JAK1 and CSF2RB, r = 0.4402, P-value =9.74e-17.


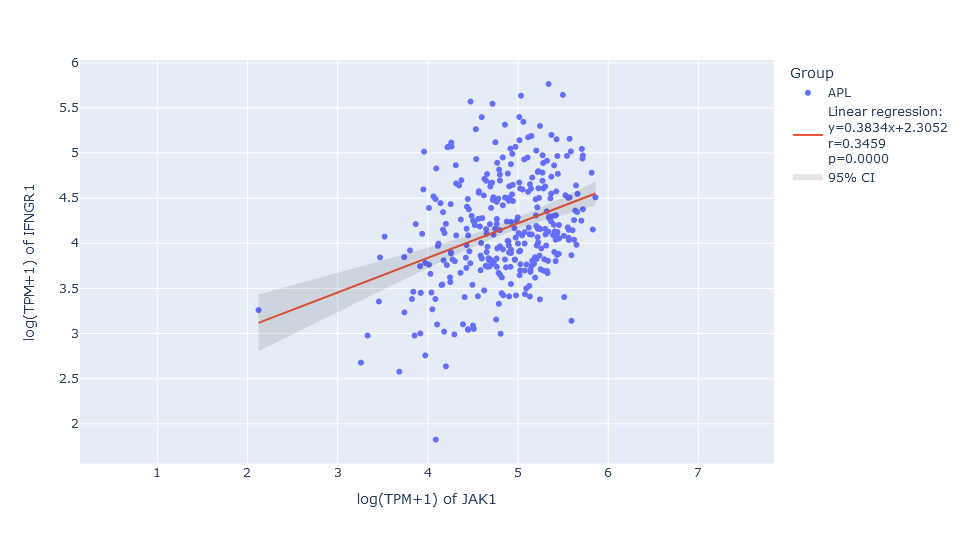


Figure S14. Correlation analysis results of expression of JAK1 and IFNGR1, r = 0.3459, P-value =1.65e-10.


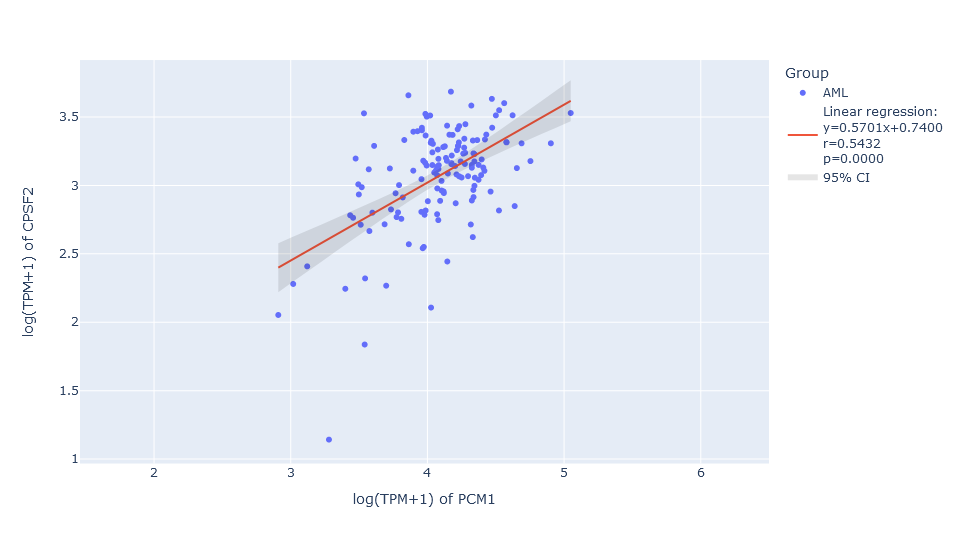


Figure S15. Correlation analysis results of expression of PCM1 and CPSF2, r = 0.5432, P-value = 2.00e-12.


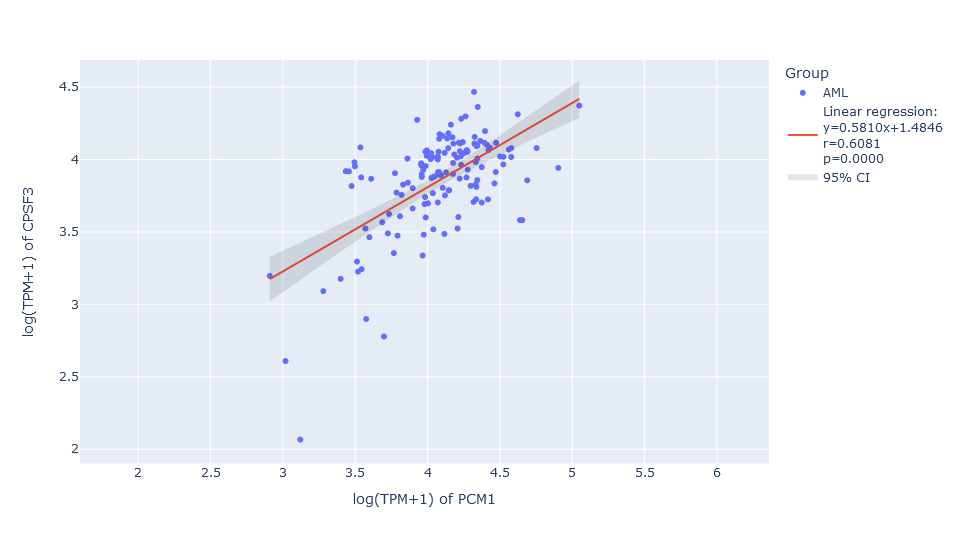


Figure S16. Correlation analysis results of expression of PCM1 and CPSF3, r = 0.6081, P-value = 6.28e-16.


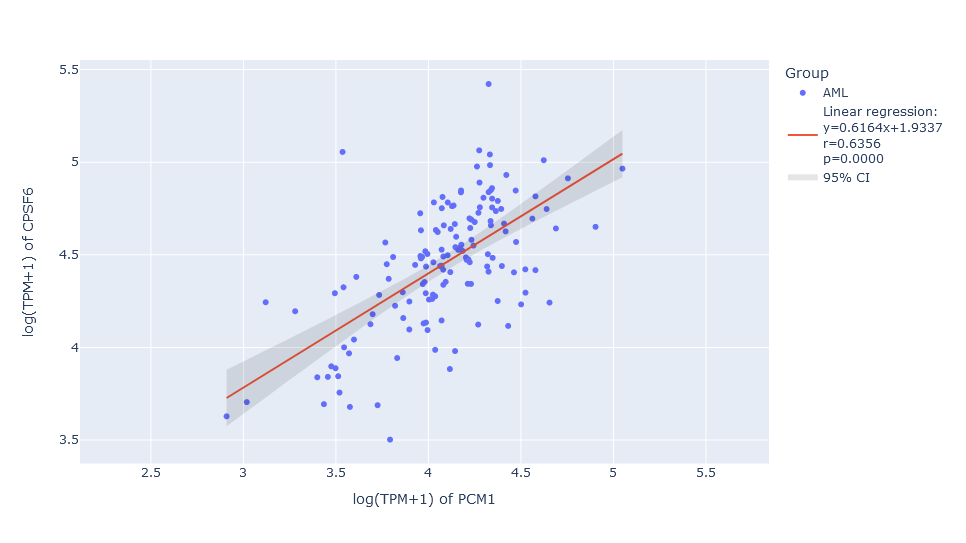


Figure S17. Correlation analysis results of expression of PCM1 and CPSF6, r = 0.6356, P-value = 1.15e-17.


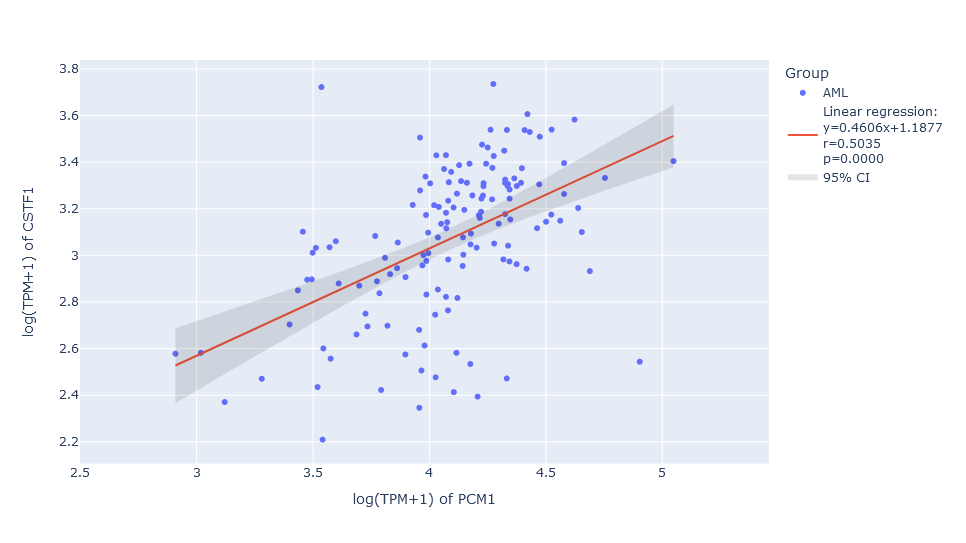


Figure S18. Correlation analysis results of expression of PCM1 and CSTF1, r = 0.5035, P-value = 1.26e-10.


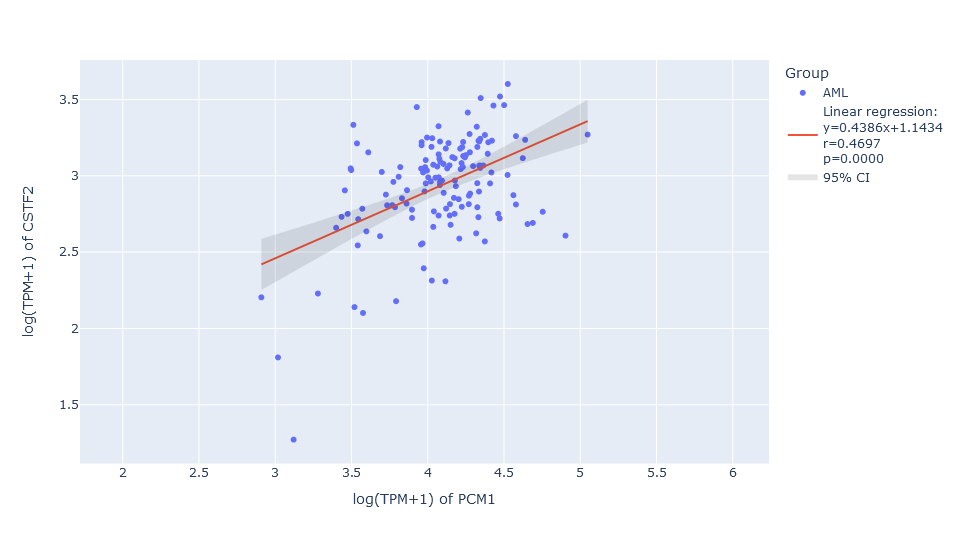


Figure S19. Correlation analysis results of expression of PCM1 and CSTF2, r = 0.4697, P-value = 2.86e-09.


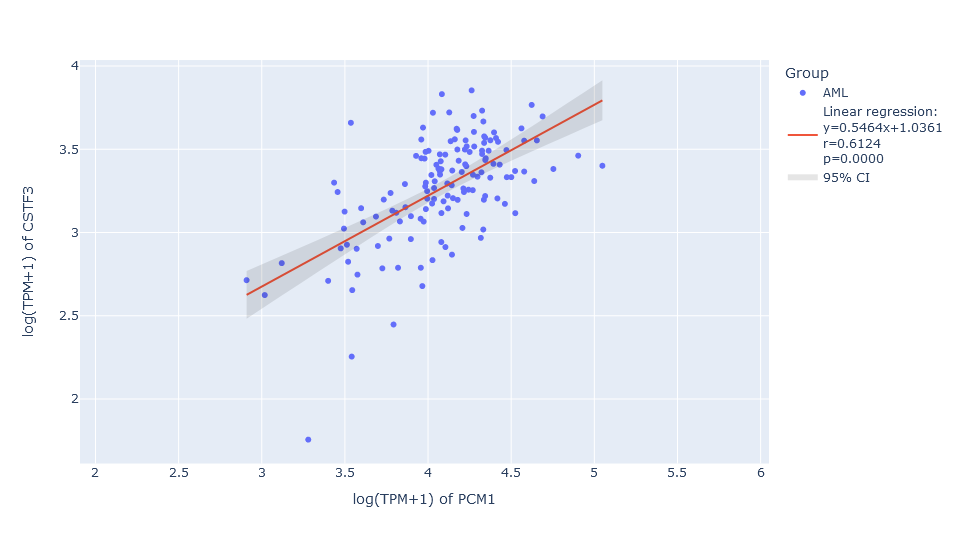


Figure S20. Correlation analysis results of expression of PCM1 and CSTF3, r = 0.6124, P-value = 3.47e-16.


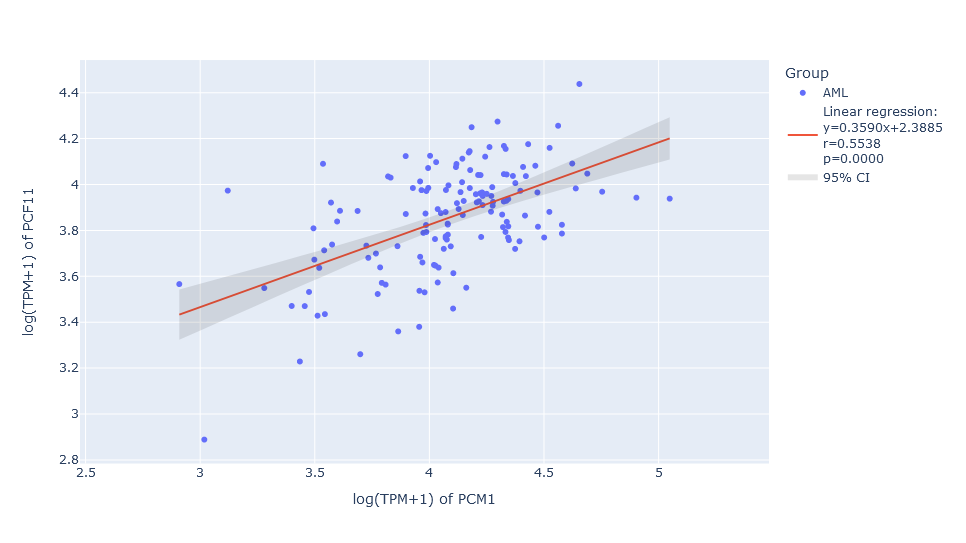


Figure S21. Correlation analysis results of expression of PCM1 and PCF11, r = 0.5538, P-value = 6.03e-13.


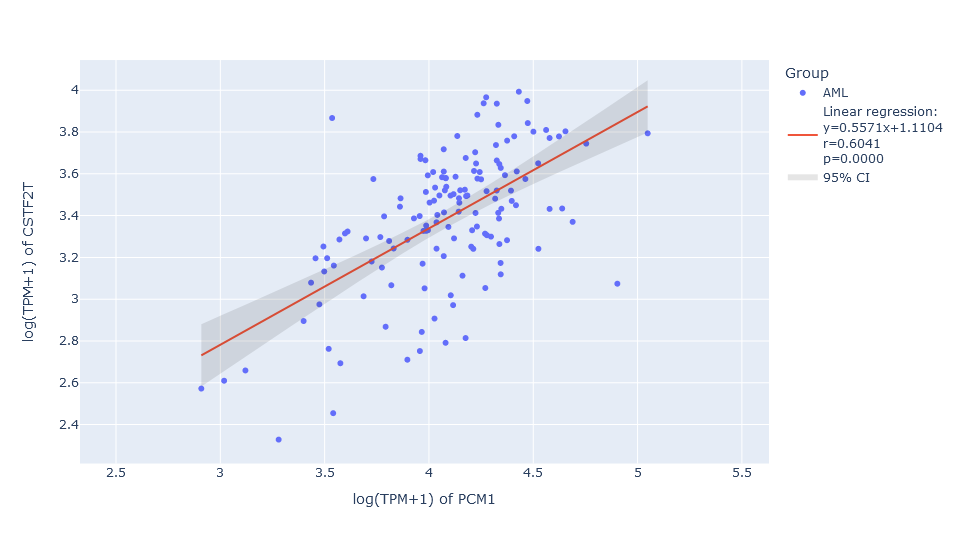


Figure S22. Correlation analysis results of expression of PCM1 and CSTF2T, r = 0.6041, P-value = 1.10e-15.


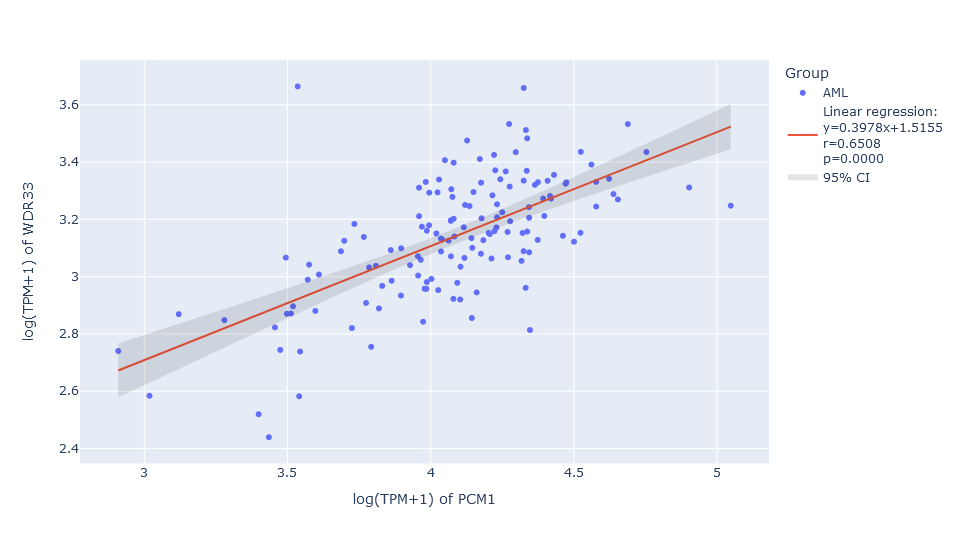


Figure S23. Correlation analysis results of expression of PCM1 and WDR33, r = 0.6508, P-value = 1.05e-18.


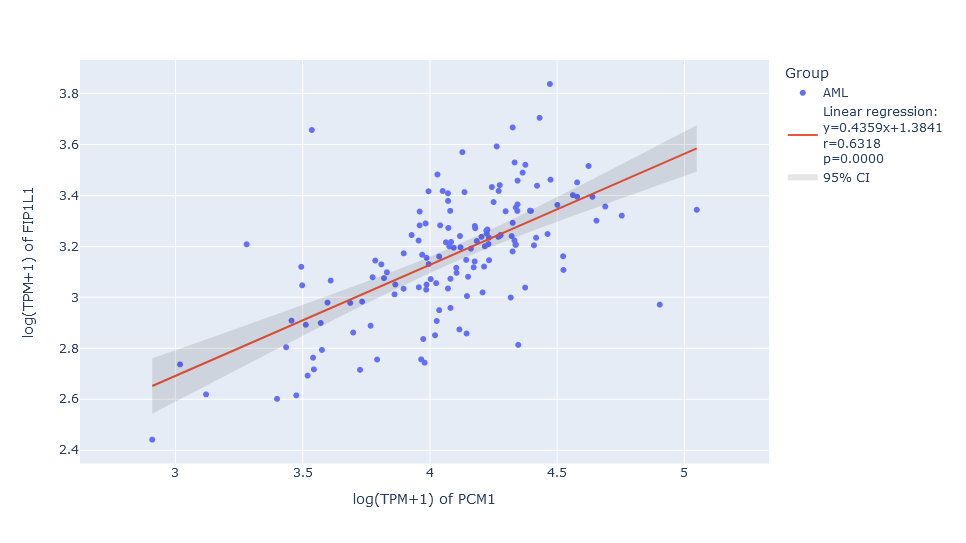


Figure S24. Correlation analysis results of expression of PCM1 and FIP1L1, r = 0.6318, P-value = 2.05e-17.


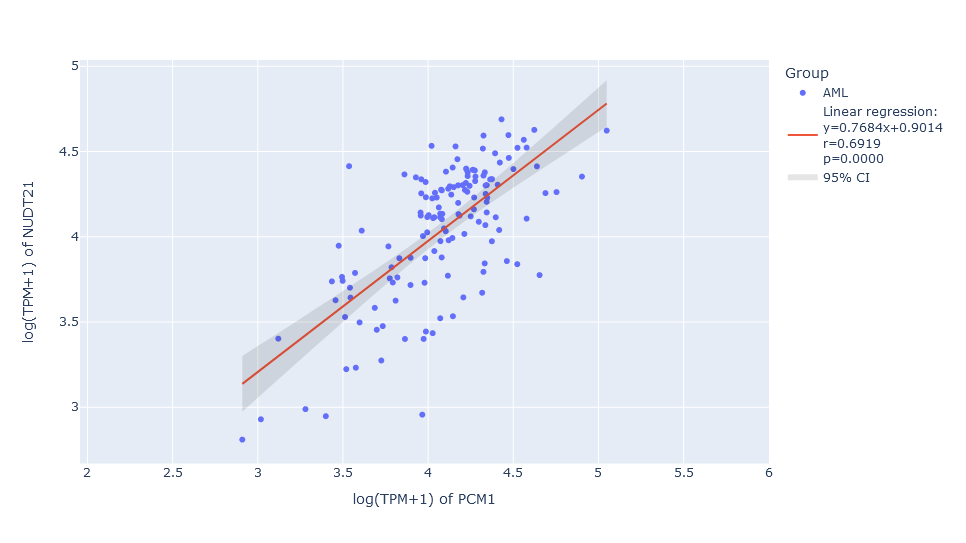


Figure S25. Correlation analysis results of expression of PCM1 and NUDT21, r = 0.6919, P-value = 7.90e-22.
